# Supplementary material for: The strength of balance: Strength and dynamic balance in children with and without hypermobility
Source: PLoS One. 2024 Jun 26;19(6):e0302218. doi: 10.1371/journal.pone.0302218 (PMC11206839; doi:10.1371/journal.pone.0302218)
Supplement: Attachment — Submitted filename: Response to Reviewers.docx [file pone.0302218.s002.docx]

| S/N | Original manuscript | Revised manuscript |
| --- | --- | --- |
| 1 | Title. Considering the explanations provided in the Introduction section and variables assessed, the title is not appropriate. Please edit it. | Thank you for raising this point. The title has been changed to reflect the context of the study.  Title: The strength of balance: Strength and dynamic balance in children with and without hypermobility |
| 2 | Please add “pain” and “dynamic balance” to the Keywords section. | Thank you for pointing this out. Pain and dynamic balanced are added to the keywords.  **Keywords:** Generalized Joint Hypermobility, Strength, Dynamic balance, Children, Proprioception, and Pain. |
| 3 | Introductions. Please insert reference/references for different sentences in the Introduction section. For some sentences, no reference was used | Thank you for raising this point. All sentences are now referenced excepts are those that are the opinions of the authors |
| 4 | Introduction, please summarize the Introduction section. | Thank you for this comment. The introduction has been summarized and the word count reduced from 1222 words to 889 words. Some sentences were adapted to ensure connection (flow) with the text after reducing the number of words |

REVIEWER 2

| S/N | Original manuscript | Revised manuscript |
| --- | --- | --- |
| 1 | The introduction in its present form is too long. It distracts the reader from the main topic. Please summarize it. | Thank you for this comment. The introduction has been summarized and the word count reduced from 1222 words to 889 words. Some sentences were adapted to ensure connection (flow) with the text after reducing the number of words. |
| 2 | Some of the sentences in the introduction have no references. Please insert them. | Thank you for raising this point. All sentences are now referenced except those that are the opinions of the authors. |
| 3 | Table 6 is inserted in the manuscript text. | Thank you for pointing this out. The insertion of Table 6 has been adapted. |
| 4 | The clinical implication of this study is added. | Thank you for this comment. This has been included in the strength and limitations section of the manuscript. “Our study has also provided data on functional tasks like stair climbing or lifting a heavy box in children with GJH, which is scarce in literature, making comparisons with other studies difficult. Clinically, tests of dynamic balance and functional strength should be included in assessment protocol because they are more related to activities of daily living. Also our study provides evidence of stair climbing as a prodromal impairments for children with GJH. In addition the items of the FSM are more cost effective and easily assessable than the HHD. The measurement of isometric strength was done with HHD, which makes it objective, but the method has its limitations. “ |
